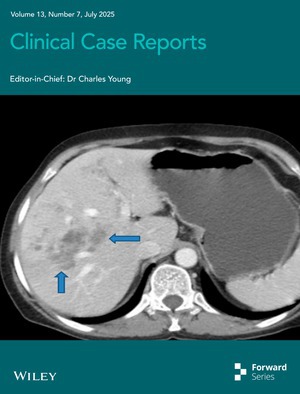# Cover Image

**DOI:** 10.1002/ccr3.70802

**Published:** 2025-08-20

**Authors:** Girma Deshimo Lema, Seife Feleke Mulatu, Zena Admasu Yferu, Getachew Bizuneh Aydagnuhm, Wogderes Bogale Gebresillassie, Yidersal Demsie Denberu, Asrat Berihun Dagnaw, Ermias Fikru Yesuf, Enguday Demeke Gebeyaw

## Abstract

The cover image is based on the article *Hepatic Fascioliasis Complicated by Multisite Venous Thromboembolism: A Rare Association and Implications for Parasite‐Associated Coagulopathy* by Girma Deshimo et al., https://doi.org/10.1002/ccr3.70647.